# Health sciences librarians of African heritage: an earnest entreaty for research

**DOI:** 10.5195/jmla.2022.1401

**Published:** 2022-04-01

**Authors:** Charles Raymond Fikar, Barbara Hallas

**Affiliations:** 1 crfikarmath@yahoo.com, Physician, Medical Librarian, Queens College, Flushing, NY; 2 bhallas@optonline.net, Health Sciences Librarian, Retired, Nassau University Medical Center, East Meadow, NY

**Keywords:** African American, minorities, diversity

## Abstract

In view of recent discussions of diversity in library work, it would seem prudent to have a good understanding of basic facts and considered opinions of health sciences librarians of African heritage concerning their career experiences, opportunities for advancement, perceptions of negative behavior in the library, experiences of bias and discrimination in the library, existence of special information needs of patrons of African heritage, and interactions with non-African-heritage medical librarians and staff. Since there is a dearth of literature and research on these topics, this commentary will attempt to stimulate and encourage such work by providing a brief summary of currently available literature and research and providing some ideas for future academic endeavors.

We will begin this commentary with two short but powerful quotations:

The plague of racism is insidious, entering into our minds as smoothly and quietly and invisibly as floating airborne microbes enter into our bodies to find lifelong purchase in our bloodstreams [1].

As libraries remain predominantly staffed and structured by the majority White culture, the few librarians of Color often find themselves feeling marginalized and without access to a supportive group of similarly diverse-minded colleagues to whom they can relate and confide [2].

Persons of African heritage (PAH) are valuable members of the health care team and contribute to all fields and specialties within the health professions, particularly including the field of health sciences librarianship. Innumerable contributions have also been made to the field of general librarianship and library science education by this group [3, 4, 5]. It is apparent from published statistics that these individuals, as well as members of other minority groups, are, very unfortunately, underrepresented in the field of librarianship, and particularly in the field of medical librarianship. Statistics compiled and published in 2012 by the American Library Association's Office for Research and Statistics and Office for Diversity documented that Latinx persons comprise 16.3% of the American population, but just 3.1% of librarians; African American persons comprise 12.6% of the population, but just 5.1% of librarians; Asian and Pacific Island persons comprise 5% of the population, but just 2.7% of librarians; and finally, Native American/Indigenous persons make up less than 1% of the population but just 0.2% of librarians [6]. Similar current statistics for health sciences librarians are difficult to obtain, but a 2017 MLA Salary Survey of 697 health science librarians documented that Latinx persons comprised 2.4% of responding medical librarians, African American persons 2.4%, Asian/Pacific Islander persons 4.6%, Native American persons 0.2%, multiracial persons 2.5%, and White persons 85.7% [7].

The duties, roles, and job descriptions of a health sciences librarian do not substantially differ depending upon a person's gender, color, ethnicity, national origin, sexuality, physical disability, religion, or other perceivable or non-perceivable characteristic(s). However, there are indeed patterns of perception and behavior, as well as some roles that can be assumed, that can greatly differ depending on the color or ethnicity, or other characteristics, of the librarian [8]. Some of these roles include recruitment of health sciences librarians of African heritage (HSLAH), role modeling and mentoring of HSLAH and students, collegial networking, encouragement and support of HSLAH and students, specifically seeking out medical information pertaining to PAH and literature written by health care professionals of African heritage, rediscovering and archiving the history of HSLAH and the many achievements made by health professionals of African heritage, and possibly perceived as being more easily approachable by PAH compared to a health sciences librarian who is not of African heritage. These attributes would apply as well to persons of any other minority group, ethnic group, or any other faction of marginalized humanity.

Although many positive changes have occurred worldwide in the area of civil rights for all peoples, there still exist today, to a greater or lesser extent, the remnants of racially or other types of prejudice-motivated aggressions, hatreds, biases, insults, and invalidations, with subtle instances of such being termed “microaggressions,” “microbiases,” "microinsults,” “microinvalidations,” etc. It is known that even the most inconspicuous, ambiguous, nebulous, and subtly disguised of microaggressions can be very detrimental to a person by creating a hostile work environment, by devaluing a person's group identity, by lowering work productivity, and by creating physical health and/or emotional health problems [9–14].

No surveys could be found involving large numbers of American medical librarians of African heritage in the published library, medical, or social sciences literature that describe their experiences or examine the presence of discriminatory or biased behavior encountered by this group. However, a few large surveys have been conducted that provide extremely useful and detailed information on minority librarians in Canada, Australia, and Great Britain (not specifically medical librarians) [15–17]. Literature written by individual HSLAH recounting their role as information providers and their personal experiences have appeared, but studies collecting data from large numbers of health sciences librarians are clearly elusive. Much information has been published about PAH in the public library and general academic library areas.

A PubMed search of the *Bulletin of the Medical Library Association* (1911–2001) using the text words “black” and “afro*” and “afri*” and “negr*”and the MeSH term “African Americans” revealed only eighteen citations, with only two actually dealing with American PAH. These articles discussed information-seeking behavior and also a hospital library in a predominantly PAH community. The same search done for the *Journal of the Medical Library Association* (2002–present) revealed fourteen articles, of which nine actually pertain to people of African heritage. Other databases were consulted, including EBSCO's Library & Information Science Source, which happened to have the very useful descriptor “African American librarians.” However, none of these articles discuss the HSLAH. There could be many reasons for the paucity of literature on these seemingly hidden or invisible professionals in the biomedical literature. For many researchers, there may simply be a lack of interest or professional awareness of PAH. Some may believe that this area of scholarly pursuit is not important, not needed, or disreputable. Perhaps there may also be fear that the investigator's career goals may be adversely affected, or that he or she will be shunned by peers. Additionally, funding opportunities may be lacking for research in this area. Finally, also of note is the possibility that any particular search engine may be a biased index to information and can distort or make invisible entire populations [10]. Appropriate controlled vocabulary, descriptors, and thesauri, as well as MeSH, Emtree, and other terms, can make finding information an easy task. Biased and nonexistent terms can make finding information difficult or almost impossible. Persons seeking information on a topic may therefore become discouraged and give up their research efforts because of a misleading impression of lack of information sources for their topic. But silence or neutrality about important societal issues should be an unacceptable position for both individuals and institutions. Are health sciences libraries and/or health science librarians tacitly and perhaps unconsciously supporting racial oppressions?

It is noteworthy that from the study of the biographies of many librarians of African heritage, one may discern certain pervading themes. Excelling at providing service to all persons in the community, particularly underrepresented peoples, as well as supporting community groups devoted to social change, is one such theme. Mentoring and recruiting other PAH into the field of health sciences librarianship is another. Community service, collecting and preserving the history of HSLAH, and documenting the contributions of health professionals of African heritage are among many other themes that are clearly evident [8, 18].

It seems that it would be extremely important and highly desirable that research be conducted with the purpose of finding information concerning career experiences, opportunities for advancement, perceptions of negative behavior in the library, experiences of bias and discrimination in the library, the role of mentorship by HSLAH, existence of special information needs of patrons of African heritage, and interactions with non-African-heritage medical librarians and staff, as well as other items of potential interest. Persons belonging to groups of people who also are subjected to discrimination, prejudice, microaggressions, or other negative behaviors, and wish to participate in the research, should be studied as well. Also of interest would be the determination of whether special information needs exist for PAH or health care providers of African heritage (as well as for other minority groups). It is known, for example, that among persons with cancer, there are significant differences between White persons and persons of African heritage concerning information needs about their specific illnesses as well as treatment concerns [19].

We wonder how many persons of African heritage are directors of medical or veterinary libraries? How many Latinx clinical medical librarians are there? How many lesbian persons work at the reference desk of community hospital libraries? How many librarians who have experienced severe physical or emotional trauma work in dental libraries? How many physically challenged catalogers work at medical school libraries? How do they cope with various work challenges, with whom can they share common experiences, how many other special people, including students, have they helped? Much scholarship needs to be done.

These are some of the reasons why we hopefully and eagerly request that such scholarly research be done, and we not too patiently await the answers to these types of questions concerning HSLAH as well as other minority groups. We believe it behooves the members of the profession of medical librarianship to find funding and carry out surveys and studies to elucidate the information needed to encourage changes in our profession that will inspire the welcoming of more diversity into our profession and our information centers, wherever they may be. The recent paper by Akers et al. in this journal is to be commended for their efforts in this direction [20].

## References

[1] Angelou M. Wouldn't take nothing for my journey now. New York, NY: Bantam Books; 1993. p. 121.

[2] Anantachai T, Booker L, Lazzaro A, Parker M, Establishing a communal network for professional advancement among librarians of color. In: Hankins R, Juarez M, eds. Where are all the librarians of color? The experiences of People of Color in academia. Sacramento: Libr Juice Press; 2015. p. 31–53.

[3] Wilkin BT, ed. African American librarians in the Far West: pioneers and trailblazers. Lanham, MD: Scarecrow Press; 2006.

[4] Danquah LE. Achievements of selected 21^st^-century African American health sciences librarians. In: Jackson AP, Jefferson JC, Nosackhere AS. eds. The 21st-century Black librarian in America: issues and challenges. Lanham, MD: Scarecrow Press; 2012. p. 121–25.

[5] Dawson A. Celebrating African-American librarians and librarianship. Libr Trends. 2000;49(1):49–87. Available from: http://hdl.handle.net/2142/8328.

[6] Cooke NA. Introduction. Libr Trends. 2018;67(1):1–7. DOI: 10.1353/lib.2018.0021.

[7] Corcoran K, ed. 2017 MLA Compensation & Benefits Survey. Chicago, IL: Medical Library Association; 2017:7.

[8] Henderson CL. The role of the Black health sciences librarian. In: Josey EJ, DeLoach ML, eds. Handbook of Black librarianship. Lanham, MD: Scarecrow Press; 2000. p. 683–96.

[9] Swanson J, Tanaka A, Gonzalez-Smith I. Lived experience of academic librarians of color. Coll Res Libr. 2018; 79(7):876–94. DOI: 10.5860/crl.79.7.876.

[10] Barr-Walker J, Sharifi C. Critical librarianship in health sciences libraries: an introduction. J Med Libr Assoc. 2019;107(2):258–64. DOI: 10.5195/jmla.2019.620. 31019396 PMC6466494

[11] Nadal Kl, Griffin KE, Wong Y, Davidoff KC, Davis LS. The injurious relationship between racial microaggressions and physical health: implications for social work. J Ethn Cult Divers Soc Work. 2017;26(1–2):6–17. DOI: 10.1080/15313204.2016.1263813.

[12] Alabi J. Racial microaggressions in academic libraries: results of a survey of minority and non-minority librarians. J Acad Libr. 2015;41(1):47–53. DOI: 10.1016/j.acalib.2014.10.008.

[13] Williams MT. Microagressions: clarification, evidence, and impact. Perspective Psych Sci. 2020; 15(1):3–26. DOI: 10.1177/1745691619827499. 31418642

[14] Dalton SD, Mathapo G, Sowers-Page E. Navigating law librarianship while Black: a week in the life of a Black female law librarian. Law Libr J. 2018;100(3):429–38. Available from: https://www.aallnet.org/wp-content/uploads/2018/10/LLJ_110n3_05_dalton_et_al.pdf.

[15] Li Y, Kumaran M, Cho A, Ly, V, Fernando S, Miller MD. 2021 Redux survey of visible minority librarians of Canada [dataset]. Scholars Portal Dataverse. 2022 [cited 15 Feb 2022]. DOI: 10.5683/SP3/F4LNZO.

[16] Ishaq M, Hussain AM. BAME staff experiences of academic and research libraries [Internet]. London: SCONUL; 2019 [cited 15 Feb 2022]. <https://www.sconul.ac.uk/sites/default/files/documents/BAME%20staff%20experiences%20of%20academic%20and%20research%20libraries_0.pdf>.

[17] Thorpe K. National survey on Aboriginal and Torres Strait Islander employment in Australian libraries: research report [Internet]. University of Technology Sydney; 2021 [cited 15 Feb 2022]. <https://read.alia.org.au/national-survey-aboriginal-and-torres-strait-islander-employment-australian-libraries-research>.

[18] Garthwait CS. African American librarians in the Far West: pioneers and trailblazers [Book Review]. J Access Serv 3(4):71–3. DOI: 10.1300/J204v03n04_06.

[19] Asare M, Peppone LJ, Roscoe JA, Kleckner IR, Mustian KM, Heckler CE, Guido JJ, Sborov M, Bushunow P, Onitilo A, Kamen C. Racial differences in information needs during and after cancer treatment: a nationwide, longitudinal survey by the University of Rochester Cancer Center National Cancer Institute Community Oncology Research Program. J Cancer Educ. 2018;33(1):95–101. DOI: 10.1007/s13187-016-1038-x. 27097806 PMC5074918

[20] Akers KG, Pionke JJ, Aaronson EM, Chambers T, Cyrus JW, Eldermire ERB, Norton MJ. Racial, gender, sexual, and disability identities of the Journal of the Medical Library Association's editorial board, reviewers, and authors. J Med Libr Assoc. 2021;109(2):167–73. DOI: 10.5195/jmla.2021.1216. 34285661 PMC8270382

